# Near-Infrared Perfect Absorption and Refractive Index Sensing Enabled by Split Ring Nanostructures

**DOI:** 10.3390/nano13192668

**Published:** 2023-09-28

**Authors:** Wajid Ali, Weitao Liu, Ye Liu, Ziwei Li

**Affiliations:** 1 Hunan Institute of Optoelectronic Integration, College of Materials Science and Engineering, Hunan University, Changsha 410082, China; wajidali@hnu.edu.cn (W.A.);; 2 Department of Civil, Environmental & Geomatic Engineering, University College London, London WC1E 6BT, UK

**Keywords:** nanostructure, surface plasmon, perfect absorber, refractive index sensing

## Abstract

Plasmonic nanostructures as narrowband perfect absorbers have garnered significant attention due to their potential applications in biosensing and environment detection. This study emphasizes the investigation of arrayed split ring nanostructures within the configuration of metal-insulator-metal (MIM) multilayers, resulting in a maximum light absorption of 99.94% in the near-infrared (NIR) spectral range. The exceptional absorption efficiency of the device is attributed to the strong resonance of electric and magnetic fields arising from the Fabry–Pérot cavity resonance. The resonant peak can be flexibly tuned by engineering the dielectric layer thickness, the period, and the geometric parameter of split rings. Remarkably, the device exhibits promising capabilities in sensing, demonstrating a sensitivity of 326 nm/RIU in visible wavelengths and 504 nm/RIU in NIR wavelengths when exposed to bio-analytes with varying refractive indices. This designed nanostructure can serve as a promising candidate for biosensors or environmental detection.

## 1. Introduction

Plasmonic nanostructures have garnered significant attention owing to their exceptional optical properties, including enhanced absorption and sub-diffraction light localization of electromagnetic (EM) waves [1,2,3]. Their use can be explored in a diverse range of applications, such as EM shielding [4,5], optical detectors [6,7], and high-frequency communication systems [8,9]. Recently, some advancements have centered on the narrowband perfect absorption of plasmonic nanostructures with tunable resonances [10,11,12,13]. Several strategies have been implemented to achieve narrowband perfect absorption, including designing metamaterials with a tunable phase and angular momentum [14,15,16], building hybrid metal-insulator-metal structures comprised of a dielectric layer sandwiched by two metal layers [17], and designing plasmonic single or cluster nanostructures [18]. These versatile approaches enable the development of a narrowband perfect absorber. Among these approaches, structures based on MIM configurations stand out due to their features of enhanced light absorption by reflection layers and tunable resonant peaks through Fabry–Pérot cavity resonance [19,20,21,22].

In a typical MIM nanostructure, the resonant absorber includes a bottom metallic reflection layer, a middle dielectric layer, and top plasmonic nanostructures, forming the Fabry–Pérot cavity perpendicular to the layer surface. Within such frameworks, the utilization of the Fabry–Pérot cavity emerges as a versatile mechanism that facilitates flexible control over both the resonant position and the spectral bandwidth [23,24]. In addition, inducing the chirality of top plasmonic nanostructures provides an alternative way to obtain polarization-sensitive light-absorption in the biosensing of chiral molecules and analytes [25,26,27,28,29,30]. To date, a series of narrowband perfect absorbers based on MIM nanostructures have been demonstrated across different frequency ranges [31,32,33]. To meet the complex requirement of biosensing or environment detection applications, the flexible adjustment of two or more absorption narrowbands is needed, which has always been a challenge in the visible and near-infrared spectral range. Usually, the multiple-wavelength-resonant nanostructures are constructed based on complex plasmonic clusters whose photonic modes are limited by the fabrication precision of nanostructures, and the flexible adjustment of two or more absorption narrowbands is difficult to be achieved. It is necessary to find simple plasmonic structure-elements, realizing the controllable adjustment of sensing peaks by tailoring the symmetry of nanostructures [11,16].

This work presents a narrowband perfect absorber consisting of arrayed Au/Al_2_O_3_ split-ring nanostructures on a bottom gold reflection layer. The device achieves a remarkable absorption intensity of 99.94% at the resonant wavelength of approximately 950 nm, which arises from the huge electromagnetic field enhancement. When breaking the asymmetric geometry, more gap modes are induced to enhance the plasmonic resonance achieving the narrowband, and also creating a dual band absorption with the engineering of main peak shifting to higher frequencies. The designed nanostructures remain insensitive to the polarization of incident light over a broad angular range and hold great potential in refractive index sensing with flexible tailoring of resonant peaks. The obtained results show that the sensitivity of our nanostructures working at visible and NIR spectra is 326 and 504 nm/RIU, respectively. Such high performance parameters suggest great potential for the application of label-free biosensing and environment detection.

## 2. Methods

Our designed plasmonic MIM structure consists of the unit cell of a gold–alumina Au/Al_2_O_3_ split ring located on top of a 150 nm thick gold film. The thickness of the gold film was optimized to obtain perfect absorption of EM waves at sensing wavelengths. The periodicity of the arrays was 500 nm in both x and y directions, denoted as P_x_ and P_y_, respectively. The thickness of the gold and dielectric layers in the split ring is denoted as t_1_ and t_2_, respectively. The inner and outer radii of the split ring are R_1_ and R_2_, respectively.

Finite element analysis in the frequency domain was conducted using the commercially available three-dimensional COMSOL Multiphysics software (Version 6.1). The simulation module incorporates the differential form of Maxwell’s equation, which was utilized to model the interaction between wave and matter as follows:

(1) $$\nabla \times \frac{1}{\mu_{r}}\left(\nabla \times E\right)-K_{0}^{2}\left(\varepsilon_{r}-\frac{j\sigma }{{\omega \varepsilon }_{0}}\right)E=0$$

where **∇** is the differential operator and µ_r_ and ε_r_ are the relative permeability and permittivity of the mediums, respectively. The corresponding propagation wave vector is defined as **K** for all mediums in the model. The angular frequency ω is defined as 2π/λ, where λ is the wavelength of the impinging waves. The electrical conductivity of the medium is denoted by σ, and $j=\sqrt{-1}$ is a complex term in the governing equation.

**Figure 1:**
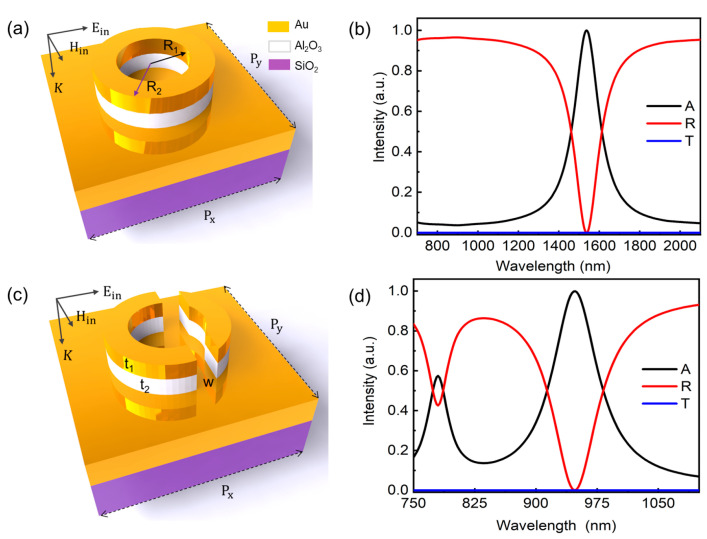
(**a**) Schematic view of a MIM unit comprising a ring. (**b**) Absorption (A), reflection (R), and transmission (T) spectra of plasmonic ring structures show a main spectral peak. (**c**) Schematic view of a split ring unit within MIM structure. (**d**) Spectra of designed split rings show two characteristic peaks by breaking the structural symmetry. The inner and outer radii are R_1_ = 80 nm and R_2_ = 130 nm, respectively. The thickness of gold and dielectric layers of the split ring are t_1_ = 35 nm and t_2_ = 40 nm, respectively, with a common cut width of w = 50 nm. The array period (P_x_, P_y_) is 500 nm.

In the context of normal incidence ($0^{{}^{\circ}}$), the incoming light wave is incident vertically to the structure surface. The direction of the electric field (**E**_in_), magnetic field (**H**_in_), and wave vector (**K**) of the incident light is aligned with the x-axis, y-axis, and negative z-axis, respectively, as depicted in Figure 1. In simulations, the boundary condition of a perfectly matched layer (PML) was applied to eliminate all scattered field interruptions. Periodic boundary conditions along x and y directions could be tailored to realize the shifting of resonant peaks arising from plasmonic coupling. Incident and receiving waveguide ports were applied at the top and bottom boundaries to obtain reflection and transmission coefficients via built-in S-parameters (i.e., S_11_ and S_21_) matrix (Supplementary Materials Section S1). The optical constants of Al_2_O_3_ and silica substrate were employed from the COMSOL materials library. The complex dielectric functions of gold were incorporated into the simulation model by utilizing the interpretation of the Drude model by P. B. Johnson and R. W. Christy, involving both the real and imaginary components of the electric permittivity [34].

## 3. Result and Discussion

The morphology of the designed nanostructures and their characteristic spectra are illustrated in Figure 1. Figure 1a shows the schematic view of a MIM unit comprising of a pair of nanorings. The associated inner and outer radii are R_1_ = 80 nm and R_2_ = 130 nm, respectively. The thickness of gold and dielectric layers of the nanostructure are t_1_ = 35 nm and t_2_ = 40 nm, respectively. Figure 1b presents the absorption (A), reflection (R), and transmission (T) spectra of the symmetric plasmonic nanostructure. It shows a near-perfect absorption for the plasmonic resonant peak at 1537 nm, which is in good agreement with similar studies [35]. The observed spectral position is located in the communication window of information, which is favorably suited for switching and optical modulation applications. However, to meet the demand of biosensing or environment detection in the NIR spectral range, the resonant peak should be shifted to near 1000 nm. It has limited abilities to tune the peak to such a large range, and can only do so by changing the structural parameters of the rings.

The schematic view of the designed split ring nanostructure is illustrated in Figure 1c. The simulation parameters can be found in the Methods section. The split ring nanostructure has a common cut width of 50 nm. In contrast to unbroken resonators [35], our designed plasmonic nanostructure in Figure 1c offers two unique features resulting from its symmetry breaking. Firstly, it enables dual narrowband absorption, significantly expanding the device’s absorption capabilities. Secondly, it provides a flexible approach to tailor the shift of plasmonic resonances towards higher frequencies (Figure 1d). These spectra are calculated under normal incidence using optimized geometrical parameters. Notably, two prominent absorption peaks are observed at 775 nm and 950 nm. With a 150 nm thick gold film, the proposed device exhibits zero transmittance and enables perfect absorption at the resonant wavelength of 950 nm, which has the relationship of A = 1 – R – T ≈ 1 − R. In the configuration of the MIM structure, a thicker bottom metallic layer plays a critical role in eliminating electromagnetic wave transmission and supporting tunable Fabry–Pérot cavity resonance (Supplementary Materials Section S2). The near-perfect absorption of electromagnetic waves is attributed to the significant electromagnetic enhancement of the Fabry–Pérot cavity mode and plasmonic coupling mode. These advancements offer the potential for the efficient absorption of electromagnetic waves, meaning they have the potential to be plasmonic element structures for various optoelectronic applications.

**Figure 2:**
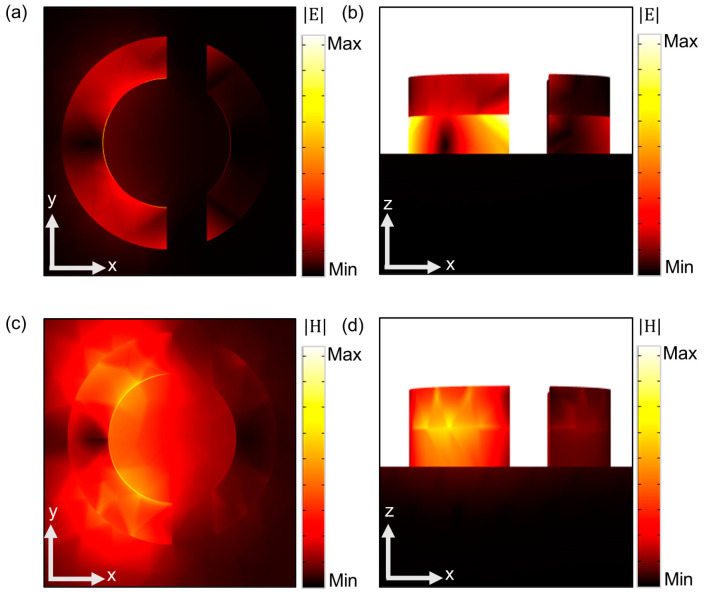
(**a**,**b**) Side and front views of electric field € distribution at the resonant wavelength of 950 nm. (**c**,**d**) Side and front views of corresponding magnetic field (H) distribution at 950 nm.

To elucidate the physical mechanism behind the characteristic modes leading to perfect absorption at the resonant wavelength, the distribution of electric and magnetic fields at the resonant wavelength was examined. Figure 2 illustrates the simulated EM field distribution at 950 nm. Figure 2a,b depict the map of EM field intensity (E) in the x-y plane and x-z plane, respectively. The energy of the EM field is primarily confined within the dielectric region due to the Fabry–Pérot cavity effects. The field distribution and intensity confirm the surface plasmonic excitation at resonant frequencies. In the MIM configuration, the current loop within the structure generates a substantial magnetic moment that interacts with the magnetic field of the incident light, leading to the formation of a magnetic resonance. This resonance arises from the coupling between the magnetic moment of the current loop and the oscillating magnetic field of the incident light [36]. The emergence of this magnetic resonance, stemming from the intricate coupling between the current loop’s magnetic moment and the incident light’s magnetic field, plays a pivotal role in augmenting the light–matter interactions within the designed structure, thereby facilitating enhanced optical functionalities. At the resonant excitation of 950 nm, the magnetic field distribution (H) at the interface of the metallic resonator and dielectric spacer are depicted in Figure 2c,d. At the interface surface of gold and dielectric layers of the split ring, the well-designed MIM structure supports the magnetic resonance and facilitates near-field enhancement. The confinement of the magnetic field plays a critical role at the resonant frequency, especially in driving the device toward near-perfect absorption.

**Figure 3:**
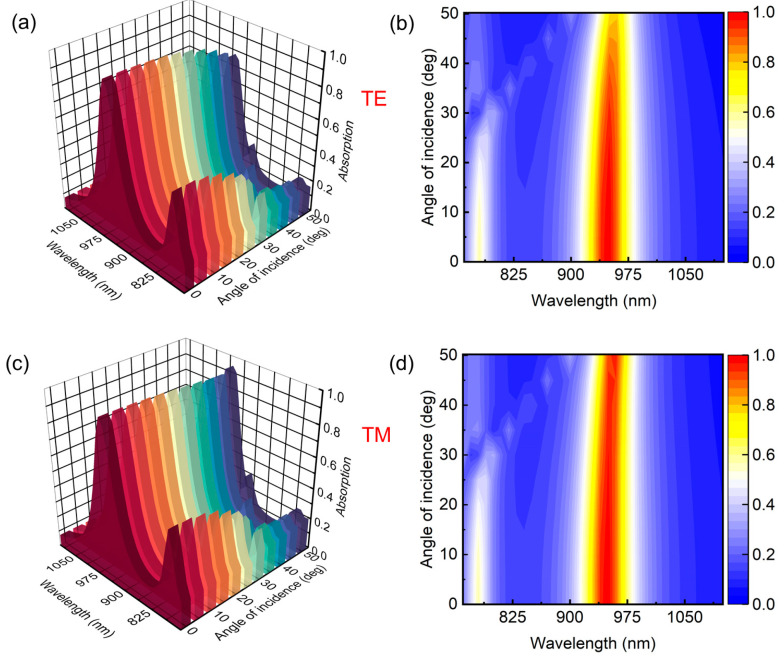
The variation of absorption spectra under the excitation with a broad range of the incident angles from $0^{{}^{\circ}}$ to $50^{{}^{\circ}}$ for (**a**,**b**) TE polarization and (**c**,**d**) TM polarization.

The asymmetric structure of the split ring significantly contributes to asymmetric field enhancement. One side of the split ring exhibits a higher field intensity compared to the other, giving rise to dual bands and less polarization-dependent absorption, as demonstrated in Figure 3. Figure 3a,b showcases the absorption characteristics of the device for transverse electric (TE) polarized waves across an incidence angle range of $0^{{}^{\circ}}$ to $50^{{}^{\circ}}$ degrees. Up to an inclination of $30^{{}^{\circ}}$, the absorption intensity remains relatively uniform. However, a further inclination of the TE incident waves results in a continuous minor decrease in the intensity and red shifting of the resonant wavelengths, as depicted in Figure 3b. In contrast to the TE polarization, the device shows relatively stable absorption intensity for a long angular range of TM-polarized light, as shown in Figure 3c,d. These results show excellent stability of periodic split rings at different incident angles.

**Figure 4:**
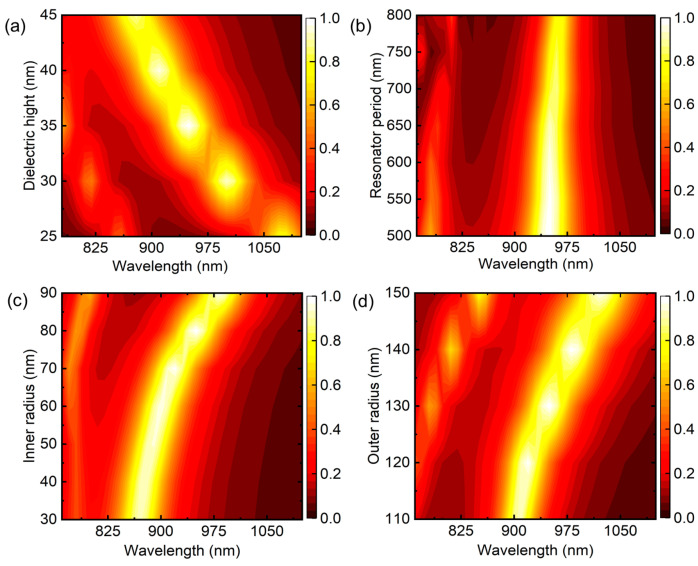
Total absorption spectra of the absorber with various structure parameters, with (**a**) various spacer thicknesses at a constant gold height of 35 nm; (**b**) varying periods of the unit cell; and (**c**,**d**) different inner and outer radii at normal incidence of EM waves.

To investigate the impact of geometrical factors on the perfect absorption feature, systematic numerical simulations of absorption spectra with varying incident angles were carried out. Figure 4 illustrates the spectral results obtained from split rings with various thicknesses, varying periodicity, and different inner and outer radii. The Fabry–Pérot cavity effect is strongly influenced by the thickness of the dielectric spacer layer in MIM-type plasmonic absorbers. As the dielectric thickness varies, it affects the phase relationship between the incident and reflected waves, leading to constructive or destructive interference at specific wavelengths. As a result, the proper investigation of this effect is crucial in plasmonic-absorber-based applications such as biosensing.

Figure 4a illustrates a map showcasing the direct correlation between the absorption intensity, resonant position, and dielectric thickness. As the dielectric thickness increases, the absorption intensity decreases, and the resonant wavelength shifts towards the blue end of the spectrum. Conversely, for thinner thicknesses, the absorption intensity increases, and the resonant wavelengths shift towards the red end of the spectrum. This behavior is attributed to the reduced interaction of charges in wide-gap metallic layers, which influences the resonant modes and absorption characteristics of the device.

In Figure 4b, the device’s intensity and plasmonic resonance response are unchanged, with a periodicity length ranging from 500 to 625 nm. However, when the separation between nanoresonators exceeds a certain threshold of 625 nm, a significant portion of the incident wavelengths encounter a bare gold film, resulting in a lower intensity of absorption but a higher intensity of reflectance. The inner radius $R_{1}$ and outer radius $R_{2}$ of the resonator have similar effects on the absorption intensity and peak resonance position, as depicted in Figure 4c,d. With an increase in the inner radius, the absorption intensity slightly increases and reaches a maximum value of approximately 99.94% for an 80 nm radius. However, when the inner radius is further increased, the absorption intensity appears to be decreased, accompanied by a redshift of the peak position towards higher wavelengths, as shown in Figure 4c. A similar trend is observed for the outer radius $R_{2}$ across the entire spectrum, as shown in Figure 4d. Notably, unlike the inner radius $R_{1}$, the resonance position exhibits a significant redshift with an increase in the outer radius $R_{2}$. This behavior can be attributed to the effective width $\left(∆R=R_{2}-R_{1}\right)$ of the resonator, which becomes more influential as the outer radius increases.

The plasmonic resonance position in the proposed plasmonic perfect absorbers exhibits a strong dependence on the surrounding environment, making them suitable for the refractive-index-based label-free detection of biomolecules, such as proteins, DNA, or explosive molecules. The performance of such sensors can be evaluated in terms of wavelength sensitivity (S) and figure of merit (FOM), which are mathematically given by S = ∆λ/∆n and FOM = S/FWHM, respectively. The terms FWHM, ∆λ, and ∆n stand for full width at half maximum of the device absorption spectrum, the change in resonant wavelength, and refractive index of the host medium, respectively. A sensing device detected with a higher FOM is considered to be a higher-performance biomolecule detector; therefore, FOM is one of the key factors for refractive-index-based biosensors. From a mathematical perspective, a more significant change in the resonant wavelength and narrowing of the absorption spectrum’s bandwidth can increase the device’s FOM.

**Figure 5:**
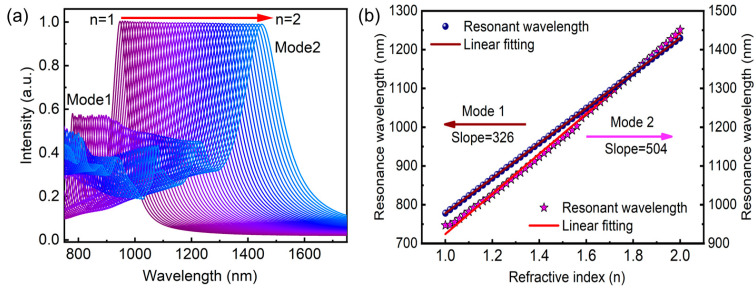
(**a**) Absorption spectra at normal incidence with refractive index (n) of the surrounding medium ranging from 1.0 to 2.0 with the increment of 0.02. (**b**) The linear fit function representation of the corresponding resonance peak position.

Figure 5 illustrates the evaluation of the absorption intensity for the device across various refractive indices of the environment, along with the corresponding peak resonance positions. In this study, air with a refractive index of 1.0 serves as the chosen reference host medium. When the environment changes to a denser medium (refractive index increases from 1.0 to 2.0), a consistent redshift in the resonance peak position is observed, as shown in Figure 5a. The wavelength shift amounts to approximately 504 nm, and these observations highlight the potential of the proposed absorber for the label-free sensitive detection and characterization of analytes based on refractive index variations.

It can be observed that the resonance position shifting to the corresponding refractive index is uniform and there are no critical changes in absorption intensity. Figure 5b shows the statistic results and fitting curves at two resonant peaks. It demonstrates the uniformity of resonance shifting by achieving a straight line with a slope of S = 326 and S = 504 for plasmonic mode 1 and mode 2, respectively. The ability of the proposed device to respond to changes in the surrounding refractive index opens up possibilities for diverse applications in biosensing, chemical sensing, and environmental monitoring. By leveraging the tunable plasmonic resonance and the associated wavelength shifts, researchers can develop highly sensitive and selective sensing platforms with promising capabilities for real-time and label-free analysis. As a result, the proposed narrowband plasmonic absorber holds great potential to be used as a probe in various biophysics and optoelectronic applications.

The process of low-cost fabrication for further applications needs to be discussed. Nanostructure fabrication is a multidisciplinary field that involves various lithographic processes to create intricate structures in the nanoscale. These fabrication techniques are essential in research fields such as electronics, photonics, medicine, and energy. For instance, electron beam lithography offers high-resolution micro-nanostructues, but it is not economical enough due to the expensive fabrication equipment and the limited area that can be fabricated at one time, making it suitable only for low-volume production and research. Nanoimprint lithography provides high resolution and throughput, but template fabrication is challenging in fabrication accuracy, especially for nanoscale structures. Among them, the photolithography technique is an alternative method for micro-nano fabrication, which has revolutionized the fabrication of integrated circuits, enabling the miniaturization of electronic devices. The processes of photolithography, followed by deposition and etching, provide a systematic low-cost strategy for high-resolution patterning and mass production in the semiconductor industry.

**Figure 6:**
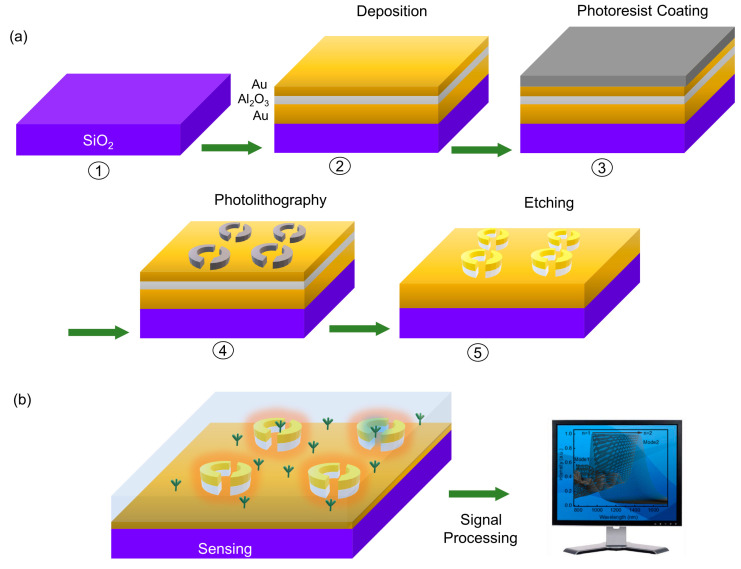
(**a**) Schematic illustration of photolithography fabrication process for preparing arrayed split-ring nanostructures. The processes involve substrate cleaning (①), deposition (②), photoresist coating (③), photolithography (④), and etching (⑤). (**b**) Schematic illustration of biosensing chip for the detection of analytes by using signal processing.

Figure 6a shows the schematic illustration of photolithography fabrication. To fabricate our designed biosensors, the SiO_2_ substrates should be flushed by cleaning solutions and prepared for next-step film deposition. Normally, organic solvent cleaning and RCA cleaning are employed to remove organic and inorganic residues, while piranha solution is selected to remove stubborn organic residues. Finally, rinsing with deionized water is necessary for the cleaning process. In the next step, three layers, gold, Al_2_O_3_, and gold again, are successively deposited to obtain the desired substrate. A target material of gold is first deposited using the sputtering technique to obtain 150 nm Au film, and then a 40 nm Al_2_O_3_ layer is grown using atomic layer deposition, ensuring a conformal and high-quality layer. Finally, a second 35 nm gold film is deposited on the top. Then, a photoresist is added using the spin-coating method, and its thickness can be regulated by adjusting the spin speed and spin time. Once the photoresist-coated substrate is prepared, it is aligned with a photomask and exposed to ultraviolet light. The light passes through the transparent areas of the mask, altering the solubility of the resist and creating a pattern. Etching and stripping are the final steps in the photolithography process. Reactive ion etching is used to etch the sample substrate, and chemical solvents such as acetone are used to remove the photoresist layer.

With the help of the low-cost fabrication of two-step large-scale photolithography and etching, our designed narrowband absorber with two plasmonic resonant peaks in both the visible and the near-infrared spectral ranges holds great potential as a biosensing chip for target molecule detection. It can be integrated with readout electronics, enabling the development of portable sensing platforms. A biosample is applied directly to the chip-based sensor, and a photodetector captures the reflected light for analysis using computer software, as illustrated in Figure 6b. The integration of electronics, miniaturization, and real-time analysis make this chip-based plasmonic absorber sensor promising for various bioanalytical sensing applications.

## 4. Conclusions

In this study, a numerically simulated simple and flexible plasmonic perfect absorber is presented. The device comprises an array of asymmetric metal-dielectric split-ring nanostructures positioned on a reflecting metallic thin film. By harnessing the magnetic resonances and Fabry–Pérot cavity effects in a MIM structure, the proposed device achieves a remarkably high absorption efficiency of up to 99.94% in the NIR spectral range. The exceptional absorption performance is attributed to the systematic modification of the nanostructure parameters, which enables optimal impedance matching. The induction of the symmetry breaking of rings helps to enhance the absorption efficiency, generate dual-band plasmonic resonance, and engineer sensing-peak shifting. Additionally, the device is insensitive to the polarization of incident light over a wide angular range. Moreover, the absorber demonstrates its potential in bio-detection by exhibiting sensitivity to a range of refractive indices, with the best value of 504 nm/RIU. With its simple structural architecture and enhanced narrowband absorption characteristics, the proposed plasmonic absorber holds promise for various applications, including in photodetection, narrowband thermal emitters, and optical modulation in the near-infrared regime.

## Data Availability

Data will be made available on request.

## Supplementary Materials

The following supporting information can be downloaded at: https://www.mdpi.com/article/10.3390/nano13192668/s1. Section S1. COMSOL simulation. Section S2. The influence of gold and dielectric layers on spectral absorption.
