# Supplementary material for: Near-Infrared Perfect Absorption and Refractive Index Sensing Enabled by Split Ring Nanostructures
Source: Nanomaterials (Basel). 2023 Sep 28;13(19):2668. doi: 10.3390/nano13192668 (PMC10574369; doi:10.3390/nano13192668)
Supplement: Supplementary file 1 [file nanomaterials-13-02668-s001.zip › nanomaterials-2631101-supplementary.pdf]

Supporting Information

Ultrahigh Near-Infrared Perfect Absorption and Refractive Index Sensing Enabled by Split Ring Nanostructures

Wajid Ali [1,†], Weitao Liu [2,†], Ye Liu [1] and Ziwei Li [1,*]

[1] Hunan Institute of Optoelectronic Integration, College of Materials Science and Engineering, Hunan University, Changsha 410082, China; wajidali@hnu.edu.cn (W.A.)
[2] Department of Civil, Environmental & Geomatic Engineering, University College London, London WC1E 6BT, UK
* Correspondence: ziwei_li@hnu.edu.cn
† These authors contributed equally to this work.

S1. COMSOL simulation

The COMSOL graphical interface offers a convenient tool for visualizing the geometry of the model. In Figure S1(a, b), the side and front view of the proposed geometry are depicted. To suppress the influence of scattered fields and minimize unwanted signal interference within the physical domain, a perfectly matched layer (PML) is implemented. The PML acts as a boundary layer that effectively absorbs outgoing waves, preventing reflections and ensuring accurate simulation results.

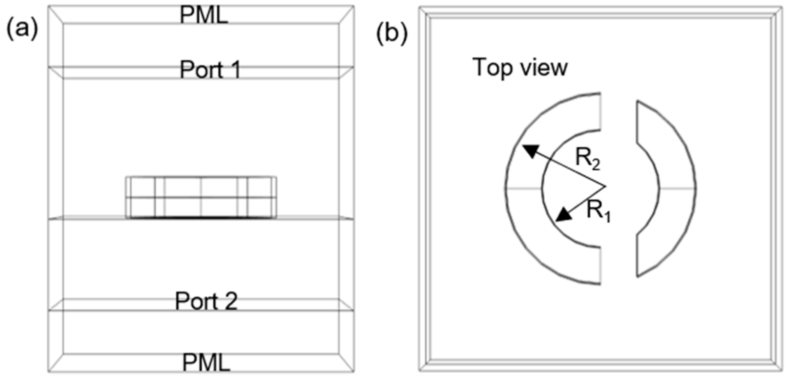

Figure S1 (a, b) Model geometry incorporated in the simulation for the proposed plasmonic perfect absorber.

S2. Influence of gold and dielectric layer on spectral absorption

To highlight the significance of the spacer in the metal-insulator-metal (MIM) configuration, the structure was initially studied without a dielectric spacer to evaluate absorption (A), reflection (R), and transmission (T), as shown in Figure S2a. The results revealed a high reflectance due to the presence of a thick gold film, which effectively covered a wide range of electromagnetic waves. To optimize the performance of the device, an alumina spacer with a thickness of 40 nm was incorporated, and various thicknesses of gold back films (ranging from 50 nm to 250 nm) were examined. Notably, Figure S2b illustrates that even with a thin 50 nm gold film, there was no significant decrease in absorption intensity. Considering the typical skin depth of gold in the visible and near-infrared range, a 150 nm thicker gold film was selected for further investigation in this study.

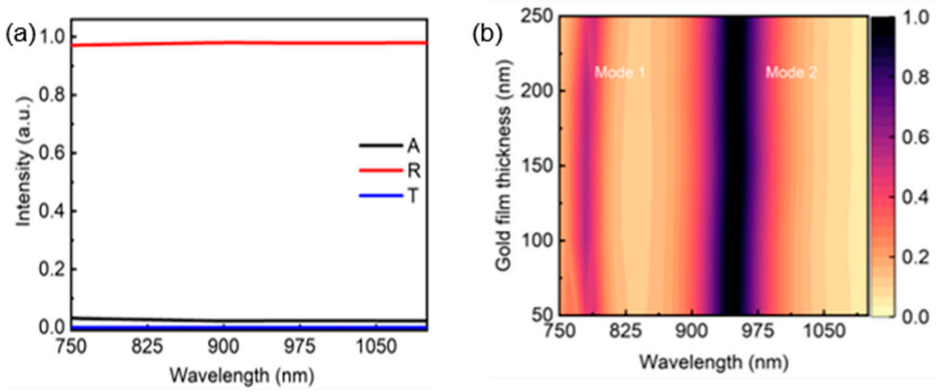

Figure S2 (a) Absorption (A), reflection (R), and transmission (T) spectra of the corresponding plasmonic structures without any dielectric material. (b) The device absorption efficiency at different gold film thickness (50 nm to 250 nm).
